# Burden of chronic diseases among sarcoma survivors treated with anthracycline chemotherapy: results from an observational study

**DOI:** 10.20517/2394-4722.2020.36

**Published:** 2020-08-07

**Authors:** Laurence H. Baker, Philip S. Boonstra, Denise K. Reinke, Erin J. Peregrine Antalis, Bradley J. Zebrack, Richard L. Weinberg

**Affiliations:** 1 Department Internal Medicine, University of Michigan Medical School, Ann Arbor, MI 48109, USA.; 2 Department of Biostatistics, University of Michigan, Ann Arbor, MI 48109, USA.; 3 School of Social Work, University of Michigan, Ann Arbor, MI 48109, USA.; 4 Division of Cardiovascular Medicine, Department of Internal Medicine, University of Michigan, Ann Arbor, MI 48109, USA.

**Keywords:** Coronary artery disease, cardiovascular disease, sarcoma, anthracycline, cancer survivor

## Abstract

**Aim:**

Cardiovascular disease is a leading cause of mortality among long-term cancer survivors treated with large total doses of doxorubicin. An increase in coronary artery disease (CAD) among childhood cancer survivors by age 45 has been observed and is driven by primarily anthracycline chemotherapy and to a lesser extent chest radiation that includes the heart in the radiation field. The risk factors and associated chronic diseases (hypertension, *etc*.) are well known for CAD and can be often prevented or treated, thus reducing the risk of CAD in these patients. We piloted a risk-based survivorship clinic in an academic medical center to characterize the distribution of risk factors for CAD and improve the quality of life in a population of sarcoma survivors treated with doxorubicin.

**Methods:**

We followed a prospective cohort of sixty-one survivors of bone and soft tissue sarcoma treated with doxorubicin chemotherapy (> 400 mg/m^2^) and at least 2 years post-therapy attending the sarcoma survivorship clinic. We collected clinical, demographic data, and patient reported outcomes via PROMIS questionnaires annually.

**Results:**

We demonstrated a high burden of chronic diseases in this population. Among six chronic conditions that are known risk factors for CAD (hypertension, diabetes, obesity, chronic inflammation, kidney disease and dyslipidemia), more than one-fourth (26%, 16/61) of patients had three or more of these risk factors at baseline visit, and 49% (30/61) had two or more.

**Conclusion:**

The results of this pilot study support the presence of modifiable CAD risk factors in this population of sarcoma survivors. Evidence-based guidelines for high-risk survivors of rare cancers are needed.

## INTRODUCTION

In 1973, the Southwest Oncology Group (SWOG) reported a phase II trial of 472 patients accrued in 17 months that demonstrated the clinical efficacy of an exciting new cytotoxic chemotherapy, doxorubicin in treating patients with advanced breast cancer, Hodgkin’s lymphoma, and multiple types of sarcomas^[1]^. SWOG further reported findings of cardiac toxicity manifested as irreversible heart failure^[2]^. In a subsequent follow-up publication, the impact of cumulative dose on long-term effects, most notably cardiac toxicity, leading to dose-limiting treatment schedules was described^[3]^. The first attempt to diminish this cardiomyopathy was also reported by SWOG in a randomized clinical trial reporting the benefit and toxicity of doxorubicin administered as a bolus injection vs a continuous infusion over 96 h^[4]^. The infusion schedule was associated with less toxicity. The importance of anthracycline chemotherapy in pediatric and adult populations was underscored, but was rapidly followed by the recognition that the long-term effects were unknown, especially in pediatric populations. The launch of the landmark Childhood Cancer Survivor Study in 1994 provided a powerful tool to account for the long-term effects of anthracycline chemotherapy on the first cohort of pediatric patients, those treated between 1970 and 1986^[5]^. Following the example of this important study, the focus has been on retrospective analysis and surveillance of the long-term effects of treatment.

Anthracycline chemotherapy and chest radiation make cardiovascular disease (CVD) a leading cause of morbidity and mortality among childhood cancer survivors^[6–9]^. Cardiomyopathy secondary to cumulative doxorubicin exposure has been a concern among oncologists, because it is often irreversible and fatal^[10]^. Over the years we have learned to prevent this cardiomyopathy by capping the dose and/or adding dexrazoxane^[11]^. More recently, the Childhood Cancer Survivor Study suggested that whereas the incidence of cardiomyopathy decreases since the time of treatment, the incidence of coronary artery disease (CAD) increases^[12,13]^. Cumulative incidence of CAD now significantly exceeds the cardiomyopathy by age 45^[13]^. CAD is also associated with doxorubicin exposure, presumably by altering the innate immune pathway and initiating CAD^[14]^. CAD is associated with several modifiable risk factors and chronic diseases including hypertension, dyslipidemia, tobacco use, metabolic syndrome, and diabetes. These chronic illnesses are most often managed by family physicians and primary internists.

Effective management of these risk factors reduces the incidence of CAD^[7]^. Exposure to anthracycline chemotherapy and radiotherapy are recognized as CVD risk factors on par with hypertension and diabetes, but are not included in cardiovascular risk prediction tools, nor is high-sensitivity C-reactive protein (hsCRP), which Ridker and colleagues have identified as a key linkage of inflammation induced by anthracycline and CAD, leaving individual practitioners to determine risk^[15–18]^. While the American College of Cardiology/American Heart Association (ACC/AHA) guidelines on the management of cholesterol and the guidelines for primary prevention of CVD recommend using the Pooled Cohort Equation to estimate cardiovascular risk in the general population^[19,20]^, this and other risk calculators do not account for anthracycline chemotherapy or chest radiation, key drivers of CAD risk in survivors of cancer, especially in younger patients. Most risk-scoring systems do not include patients at or younger than age 40, which is concerning as this is precisely the population of cancer survivors that demonstrates manifestation of CAD. Chow *et al*.^[12]^ developed a prediction model to account for treatment-related risk factors and age at diagnosis based on 5-year cancer survival, but did not include hypertension, dyslipidemia, and diabetes. Conventional risk factors (obesity, hypertension, dyslipidemia, and diabetes) have been characterized by some as non-cancer related issues^[21]^. However, we now have evidence that obesity, hypertension, diabetes and lipid abnormalities can and do result from cancer therapies. The adolescent who has a lifelong disability from an amputation-sparing knee reconstruction should expect decreased mobility and an increased risk of chronic pain, depression, and obesity as a consequence of cancer diagnosis and care. In the absence of guidelines for the timing and frequency of surveillance of chronic conditions and management of CAD risk factors, oncology providers are left with inadequate knowledge about (what may be perceived as) non-oncology care, and primary care providers are left with inadequate knowledge about cancer-specific follow-up care^[22]^.

Adult survivors of childhood cancers, especially those treated with anthracycline chemotherapies, are at a much higher risk of developing chronic conditions than other cancer populations^[5,12,13]^, making the years post-treatment a crucial window of opportunity to diagnose and manage chronic health conditions. Survivors of osteosarcoma and Ewing sarcoma have a 39-fold greater risk of acquiring severe, life-threatening, and even fatal chronic disease(s) than their siblings^[5]^. However, adherence to long-term follow-up among adolescent and young adult survivors of childhood cancers sharply declines after treatment ends, with primary care filling the healthcare gap for survivors of childhood cancers^[23]^. A 2014 survey of general internists, published in the *Annals of Internal Medicine*, queried their comfort level and preferences for caring for survivors of childhood cancers^[24]^. A sizeable minority of respondents reported being “somewhat comfortable” or “comfortable” caring for Hodgkin’s lymphoma, acute lymphoblastic leukemia, and osteosarcoma patients (36.9, 27.0 and 25.0%, respectively), presumably due to the rarity and treatment complexities of these diseases. A similar survey of 2,520 family physicians in the United States and Canada confirmed that physicians were equally uncomfortable caring for survivors of Hodgkin’s lymphoma, acute lymphoblastic leukemia, and osteosarcoma^[25]^. This study further revealed that 81% of respondents had cared for two or fewer survivors of childhood cancer in the preceding five years! It is reasonable to posit that the majority of primary care physicians, including both pediatricians and internists, will never see a patient with osteosarcoma in their practice. Knowledge of rare cancers accumulates with the experience of the oncologist, and long-term care for survivors is no different. Thus, a model of care tailored to the needs of these patients is necessary to prevent and manage chronic late effects.

To move the study of survivorship care forward we sought not to replicate the model of surveillance of the long-term effects of anthracycline chemotherapy, but to actively intervene and treat those long-term survivors for dyslipidemia, hypertension, obesity, diabetes, anxiety and depression. This model was created in recognition that the patients being monitored for recurrence were developing chronic conditions which were not being addressed in part due to lack of knowledge from primary care physicians or a paucity of care, such as in the mental health care field. Recognizing that a “one size fits all” approach will not adequately serve the heterogeneous population of cancer survivors, we piloted a survivorship clinic for survivors of bone and soft tissue sarcomas led by a medical oncologist^[26]^. This prospective cohort study was conducted to identify and treat risk factors for CAD among adult survivors of sarcoma, including those diagnosed as children, adolescents and young adults, and adults, as sarcomas occur at all ages. Risk factors for CAD are well described and often modifiable^[14,19]^. We share preliminary data from the fifth year of this prospective cohort study confirming a significant burden of chronic diseases in this population.

## METHODS

### Study population

To be eligible for enrollment in the survivorship clinic, survivors had to be 18 years or older at time of first visit, be at least two years past active chemotherapy (adjuvant or neoadjuvant doxorubicin), and be willing to return for an annual visit. The first enrollment was in October 2014 and the most recent was in May 2019. Survivors could be either self- or provider-referred and did not have to have received treatment at the host institution. Our study was approved by our institution’s Institutional Review Board (HUM00095825), and informed consent was obtained from all patients. Patients who did not consent to participate in the prospective cohort study were not included in this analysis but are still seen in the clinic.

### Risk-based survivorship clinic

In the absence of existing clinical guidelines for the care of this population of high-risk survivors of sarcoma, consultation was sought from nephrology, cardiology, endocrinology, lipidology, psycho-oncology, physical medicine, and nutrition in development of the clinical protocol with a focus on laboratory surveillance for the chronic diseases. Biometric and laboratory data were collected to encompass the conventional cardiovascular risk factors of hypertension and elevated total cholesterol, as established by the Framingham Heart Study^[27]^, obesity and high-sensitivity C-reactive protein hsCRP as found in the Reynolds Risk Score^[28]^, and diabetes as advocated by the ACA/AHA^[20]^. The Multi-Ethnic Study of Atherosclerosis risk calculator includes coronary artery calcium scores, which will be collected going forward^[29]^. Importantly, while the Pooled Cohort Equation can calculate a lifetime risk score for persons aged 20–59 years, no risk assessment is intended to accurately predict risk in individuals under age 30 and certainly not with a history of anthracycline chemotherapy and/or chest radiation.

At the enrollment visit and annually thereafter, patients received a comprehensive health examination with a focus on cardiovascular illness, patient-centered surveillance based on past therapy, family history, genetic predispositions, lifestyle behaviors, and comorbid health conditions. Patients were assessed for musculoskeletal dysfunction, metabolic syndrome, diabetes mellitus, hypertension, cardiac diseases, anxiety/fear disorder, depression, renal insufficiency, and obesity. Treatment history, demographic, family history, biometric, and patient risk assessment data were also collected at each visit. The patient’s risk assessment was reviewed with the nurse practitioner, who engaged the patients in education about their risks and counseled them on lifestyle changes to reduce their risk of chronic illness, such as exercise, weight reduction, nutrition modification, and mindfulness. Surveillance for disease recurrence was personalized to the primary sarcoma. Clinic visits averaged 60+ min of face-to-face interaction with the clinician. The nurse practitioner prepared the initial draft of the survivorship care plan after each annual visit, which was shared with the patients and their providers. The survivorship care plan included laboratory test results, imaging studies, treatment history, a cancer screening schedule, medication and vaccination review, and management plan. About 1.5–2.5 h were spent on pre-visit patient care and after-visit documentation and in the creation of the survivorship care plan.

### Data collection

Patients annually completed a health questionnaire that included family history, fertility, behavior, patient risk assessment, and patient-reported outcome measures, the results of which would be reported in a subsequent paper. We used the Patient-Reported Outcomes Measurement Information System (PROMIS), developed by the National Institute of Health (NIH) to collect PROs using PROMIS Anxiety Short Form 8a v1.0, Depression Short Form 8a v1.0, Sleep Disturbance Short Form 6a v1.0, Pain Interference Ca Bank v1.1, Physical Function Ca Bank v1.1, and Global Health Scale v1.2. Patients completed the health questionnaire via an electronic link prior to each clinic visit to support clinical care, and the data were collected and managed using Research Electronic Data Capture (REDCap) tools hosted at University of Michigan,^,^ a HIPAA-compliant web-based data capture application and database^[30]^. Biometric data and imaging were included as standard of care. Laboratory and clinical data were extracted from the patient’s electronic medical record and entered into the REDCap database.

### Statistical analysis

Clinical and laboratory measurements from each annual visit were compared to reference ranges to determine whether the measurement would be considered “abnormal”. The distributions and co-occurrences of abnormal hsCRP (≥ 2.0 mg/L), high body mass index (BMI > 30 kg/m^2^), and previously diagnosed risk factors (specifically, prior clinical diagnosis of type II diabetes, cardiovascular disease, hypertension, renal insufficiency, or high cholesterol) were graphically summarized. Statistics were stratified by age at enrollment: less than 40 years old (“18–39”) or at least 40 years old (“≥ 40”). No formal statistical inferences were conducted, due to the exploratory nature of the study and the limited sample size.

## RESULTS

A total of 61 patients had an enrollment (“baseline”) visit between October 2014 and May 2019. Among these, 43, 24, 12 and 3 patients had a total of one, two, three, or four subsequent annual follow up visits, respectively. Sixteen patients are no longer followed due to: sarcoma recurrence (*n* = 2); a new primary cancer (1); patient withdrawal due to geographic relocation, time barriers, or loss of insurance (8); physician withdrawal (1); other loss to follow-up (3); or death (1). Among 45 actively enrolled patients, 6 enrolled within 12 months of the current data snapshot, meaning that the maximum possible number of patients with at least one annual follow-up visit was 49 (43 recorded plus 6 potential).

Baseline patient characteristics, including clinical and laboratory data, are summarized in Table 1. The median age at first sarcoma diagnosis was 24 years (range 2–67), and the median age at baseline clinic visit was 42 years (range 18–82). Twenty-eight patients (46%) were between the ages of 18 and 39 at the time of the first clinic visit and 64% (*n* = 39) were diagnosed at age 39 years or younger. All patients received doxorubicin adjuvant chemotherapy (by definition of our study population), 89% had cancer surgery, and 49% received external radiation (adjuvant, neoadjuvant). The median number of years between diagnosis and baseline visit was 15 years (range 4–42).

Laboratory measurements taken at each visit (baseline, one year, and two years) that fell outside the corresponding reference range and were therefore considered abnormal are summarized in Table 2. Excluding missing values, the measurements with the largest percentage of abnormal values were hsCRP, with 51% (29/57) having a measurement of 2.0 mg/L or greater at baseline, and creatinine clearance, with 45% (25/55) having a measurement below 88 mL/min. Among the patients with a 1-year (12-month) or 2-year (24-month) visit, the percentages of abnormal hsCRP measurements increased slightly to 61 and 57%, respectively, and the percentages of abnormal creatinine clearance measurements increased slightly to 48 and 47%, respectively.

**Figure 1.**
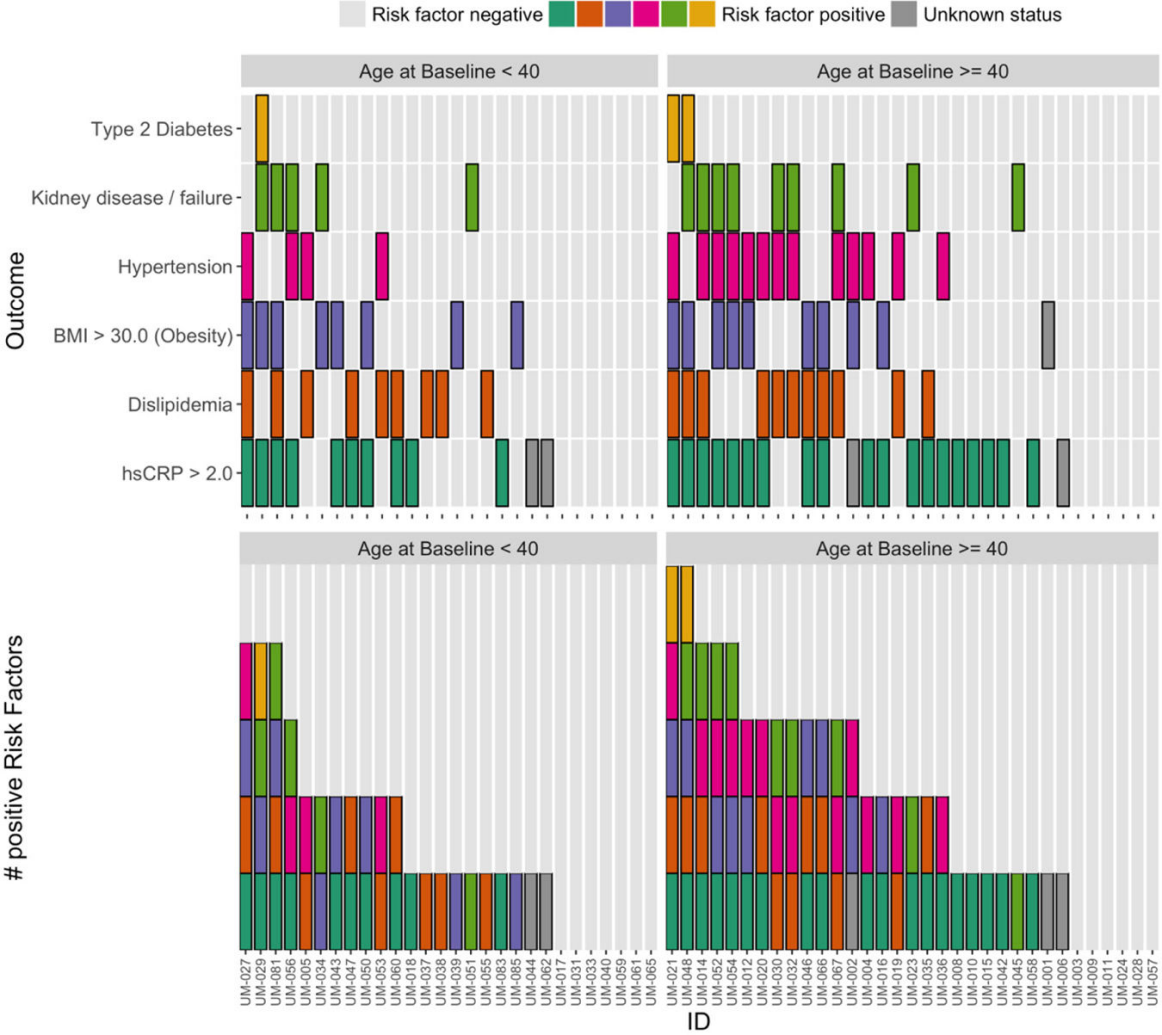
Types (top panel) and numbers (bottom panel) of risk factors identified prior to enrollment by patient, grouped by age at baseline. Note: except for body mass index (BMI) and high-sensitivity C-reactive protein (hsCRP), these are distinct from, and derived using different data than, the baseline laboratory measurements

**Figure 2.**
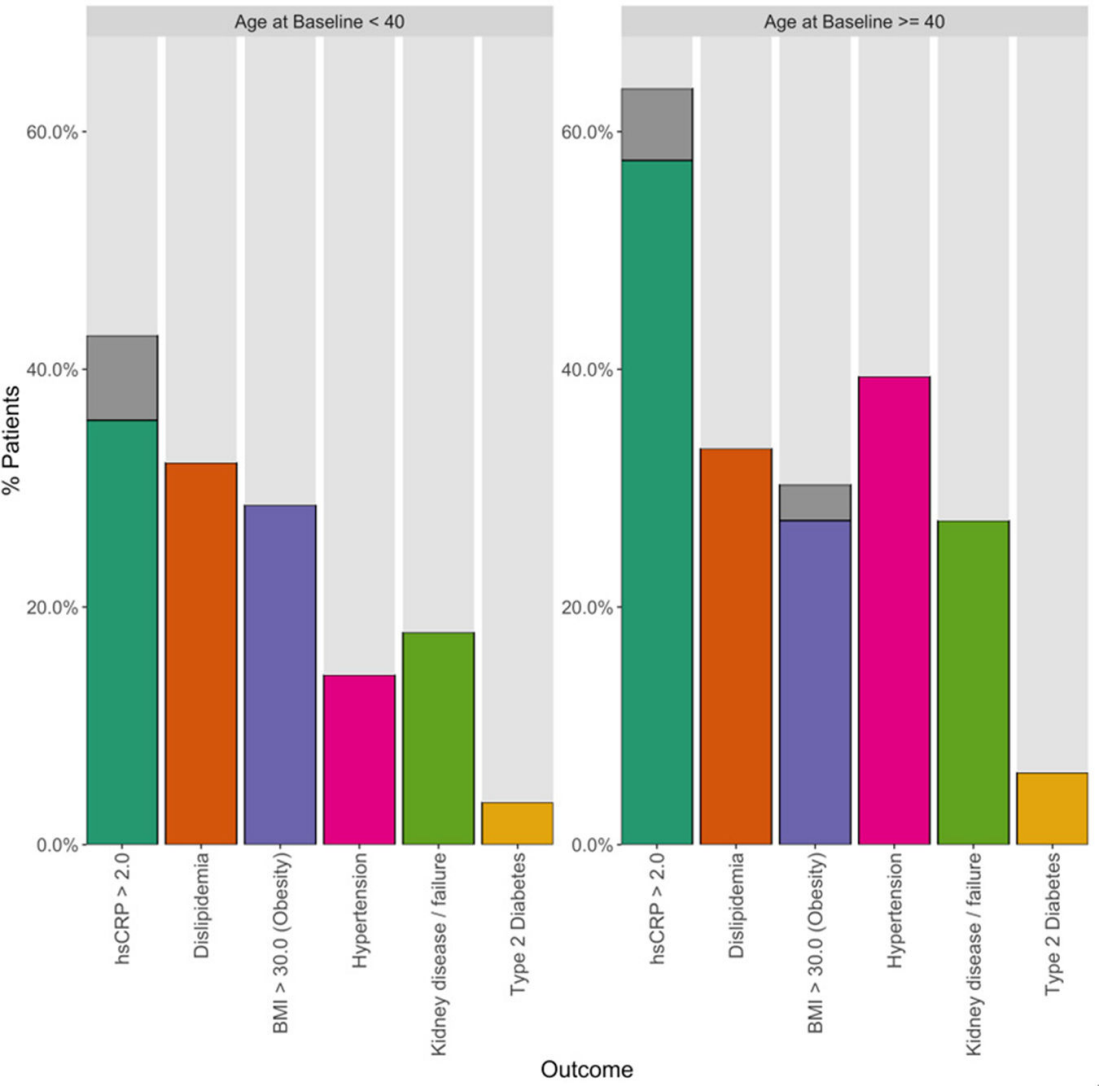
Percentages of individual risk factors identified at enrollment across all patients, stratified by age at baseline. Note: except for body mass index (BMI) and high-sensitivity C-reactive protein (hsCRP), these are distinct from, and derived using different data than, the baseline laboratory measurements

**Figure 3.**
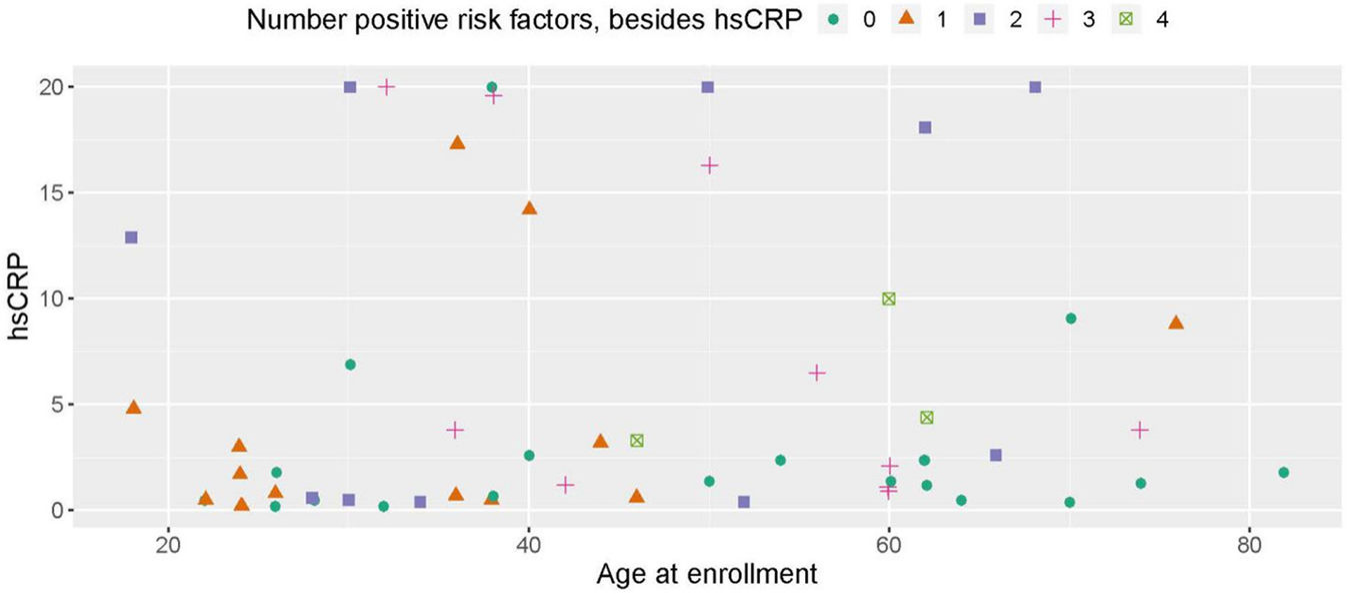
Distribution of high-sensitivity C-reactive protein (hsCRP) measurement by age and positive risk factors

Figures 1 and 2 plot the baseline distribution of risk factors for major cardiovascular events, stratified by age group. From Figure 1, 16 (26%) patients had at least three risk factors, and 30 (49%) had at least two. Nineteen patients aged 40 or older (58%) had at least two CVD risk factors identified, and 12 (36%) had three or more risk factors. In 9 out of these 12 patients, one of the contributing risk factors was hsCRP ≥ 2.0 mg/L. Among patients aged 18–39, 11 (39%) had at least two CVD risk factors identified, and four (14%) patients had three or more risk factors. In all four of these patients, one of the contributing risk factors was hsCRP ≥ 2.0 mg/L. Figure 2 indicates that the prevalence of high BMI, high total cholesterol, and type 2 diabetes was comparable between patients aged 18–39 and ≥ 40. Among patients aged 18–39, hypertension was present in 4 (14%) patients. The biomarker hsCRP, indicative of inflammation and a recognized CAD risk enhancer^[20]^, was recorded in 26 patients aged 18–39; in 10 of these patients (38%), it was measured at levels > 2 mg/dL, and in 6 patients (23%), it was measured at levels > 10 mg/dL, presented in Figure 3.

## DISCUSSION

Retrospective cohort studies of childhood cancer survivors have shown that exposure to anthracycline chemotherapy and external radiation that included the heart in the field at a young age are compounded by conventional risk factors, such as obesity, hypertension, and lipid disorders^[5,7]^. A retrospective study of adult cancer survivors similarly identified poorer overall survival for patients who subsequently developed CVD as well as a relationship between therapeutic exposure and the development of CVD^[31]^. Our cohort of sarcoma survivors treated with moderate dosing of anthracyclines confirms the presence of multiple CAD risk factors, beyond what would be expected in a healthy population, particularly so for patients younger than 40. The presence of these chronic conditions demonstrates the need for active prevention and management of late effects, in addition to monitoring for relapse. This clinic was piloted solely for survivors of bone and soft tissue sarcomas treated with anthracycline chemotherapies.

Patients are educated about their risks of developing chronic conditions as a consequence of their sarcoma treatment. Our patients are counseled on behavioral and lifestyle approaches to reduce these risks, and referred to needed medical services such as psycho-oncology when available or a social worker, nutritional counseling, and physical medicine to decrease pain and improve mobility (especially for those who have had limb salvage surgery). Patients who present with a systolic blood pressure > 130 are urged to obtain a home blood pressure cuff and taught to take measurements in accordance with best practices (twice in the morning). Patients are encouraged to report their blood pressure readings electronically with the provider to accurately assess their blood pressure in acknowledgement that some patients experience transient elevations in oncology settings. Going forward, patients will have received a coronary artery calcium score to monitor for coronary artery calcification in lieu of chest X-ray alone. Surprisingly, in our series, three patients aged 18–24 were found to have visible coronary artery calcifications on routine chest computed tomography as part of their malignancy surveillance. We have learned from cardiologists that CAD is at least in part an inflammatory process. We routinely measure hsCRP and more than half of our patients had maintained an elevation of hsCRP; Isolated elevations are most often the consequence of infection. Experimental evidence demonstrates inflammatory changes soon after doxorubicin administration^[32]^. All patients with abnormal renal function are cautioned about the use of contrast agents for imaging and ibuprofen for pain management.

All patients are given a survivorship care plan and instructed to share their care plan with their other providers. Staffed by a medical oncologist and nurse practitioner, considerable time is dedicated to extended clinic visits, pre-visit patient care and post-visit documentation and creation of the survivorship care plan. First visit preparation, face-to-face evaluation, and discussion routinely extend beyond 90 minutes, though visit duration decreases with subsequent visits. We monitor the patient for increased awareness of risk factors and are pleased to see improvement in many patients.

We suggest that patients with bone sarcoma and soft tissue sarcomas exposed to anthracycline chemotherapy are best followed within a medical oncology setting. Medical oncologists have the expert knowledge of sarcoma and sufficient familiarity of survivorship literature as well as the skills in the principles and practice of internal medicine. For example, owing to the widespread use of tyrosine kinase inhibitors oncologists have had to learn again the medical management of hypertension. Management of these complex patients requires knowledge of the patient’s treatment history and associated complications, management of the suite of chronic conditions that develop over time, and a foundational relationship between the provider and the patient. It may be necessary to share these responsibilities with a willing primary internist experienced in aggressive management of lipids, hypertension, diabetes, and obesity with clear pathways of care coordination and communication. We have learned at our institution that those trained in Med-Peds are most often receptive to a shared care model.

Benefits to these survivor patients not only can be measured in quality of life but also lifespan. Prevention of catastrophic chronic medical conditions with active management strategies clearly benefits society as well. The cost in time and resources in providing this care is challenging in the current climate of minimizing return visits within oncology, reducing visit times, the utilization of extenders to conduct follow-up care, and transiting patients to primary care for management of their chronic diseases. Medical educators should be encouraged to modify medical or Med-Peds curricula to accommodate this growing societal need as the population of cancer survivors continues to grow. Experts predict that there will be more than 20 million cancer survivors by 2025. The cost of these practices to the patients as observed in our clinic is that patients are not made aware of their risks of developing chronic diseases, are unaware that they should be monitored for chronic diseases following treatment, especially at a young age, and are not having their chronic diseases managed by anyone. The frequency of risk factors in younger survivors of our clinic suggests that there is a window of opportunity to have a great impact on quality of life and longevity among patients already burdened by the experience of cancer at a young age.

We have demonstrated a burden of chronic diseases in a subset of this young population. Evidence-based guidelines are necessary to strengthen the care of sarcoma survivors but not currently offered by the National Comprehensive Cancer Network or any other body. The development of guidelines to care for sarcoma survivors has not been supported by the National Institute of Health in medical oncology. However, NIH support is increasing in pediatric oncology and so is significant support of survivorship issues in more common cancers such as breast, colon, and prostate. We recommend the continuation and expansion of this prospective study to identify and characterize risk factors and to establish guidelines for sarcoma survivors and others treated with anthracyclines. Our cohort provides rich data characteristic of a prospective study. Oncology-based survivorship care for survivors treated with anthracycline chemotherapy necessitates addressing systemic barriers within the current health system. Recognizing that some of the data are not consistent with other data presented on adolescent and young adult survivors only underscores the relevance of disease and treatment specificity when caring for cancer survivors.

Time and resources are needed to expand this model of care to cancer survivors treated with anthracycline chemotherapy. However, the cost to the growing population of cancer survivors will arguably be higher still in terms of the chronic disease burden and overall loss in quality of life. As the survivorship population grows, so will the healthcare burden of unchecked development of chronic diseases within this heterogeneous population. Our obligation to the patients does not end with the pronouncement of cure but continues to shepherd the patient to a healthy future.

## Figures and Tables

**Table 1.** Summary of patient characteristics at first clinic visit (baseline)

Demographic characteristics	Median (IQR) prevalence (*n*)	Range	Missing
Age (at diagnosis)	24.0 (14.0, 48.0)	(2.0, 67.0)	0
Age (at enrollment)	42.0 (30.0, 60.0)	(18.0, 82.0)	0
Age ≥ 40 (at enrollment)	54% (33/61)		0
Years DX to enrollment	15.0 (10.0, 20.0)	(4.0, 42.0)	0
Female sex	59% (36/61)		0
BMI (kg/m^2^)	26.9 (24.1, 30.8)	(15.9, 46.5)	1
Smoked, ever smoked	30% (17/56)		2
Alcohol, current use	57% (33/58)		0
Drugs, current use	2% (1/54)		4
Exercise, currently exercise	53% (31/58)		0
Treatment history			
Chemotherapy	100% (61/61)		
Radiation to primary site	49% (30/61)		0
Surgery	89% (54/61)		0
Chemotherapy and radiation	51% (31/61)		0
Clinical lab values			
ALT (IU/L)	21.0 (16.0, 34.5)	(9.0, 65.0)	9
AST (IU/L)	23.0 (20.0, 29.0)	(14.0, 46.0)	9
LDL (mg/L)	85.0 (75.0, 102.5)	(31.0, 175.0)	3
HDL (mg/L)	56.0 (47.0, 73.0)	(29.0, 102.0)	2
Total cholesterol (mg/dL)	169.0 (156.0, 193.5)	(98.0, 266.0)	2
Triglycerides (mg/dL)	119.0 (74.0, 160.0)	(36.0, 269.0)	2
Glucose (mg/dL)	93.0 (83.5, 100.0)	(61.0, 154.0)	2
hsCRP (mg/L)	2.1(0.6, 6.9)	(0.2, 20.0)	4
Creatinine (mL/min)	91.0 (73.0, 112.8)	(41.0, 179.0)	3
Protein/creatinine ratio (mg/g)	77.0 (61.5, 99.0)	(2.6, 161.0)	9
SBP (mmHg)	119.0 (108.0, 135.0)	(85.0, 182.0)	0
DBP (mmHg)	67.0 (60.0, 76.0)	(42.0, 97.0)	0

BMI: body mass index; ALT: alanine aminotransferase; AST: aspartate aminotransferase; LDL: low-density lipoprotein cholesterol; HDL: high-density lipoprotein cholesterol; hsCRP: high-sensitivity C-reactive protein; SBP: systolic blood pressure; DBP: diastolic blood pressure

**Table 2.** Patient frequency of abnormal readings at baseline, 12 months, and 24 months after enrollment

Variable	Normal Range	Abnormal (%), Baseline	Abnormal (%), 12M	Abnormal (%), 24M
BMI (kg/m^2^)	≤ 30	28% (17/60)	18% (6/34)	27% (6/22)
ALT (IU/L)	≤ 30	35% (18/52)	23% (7/31)	14% (3/21)
AST (IU/L)	≤ 35	10% (5/52)	6% (2/31)	5% (1/21)
LDL (mg/L)	≤ 135	9% (5/58)	6% (2/33)	10% (2/21)
HDL (mg/L)	≥ 40	15% (9/59)	3% (1/33)	10% (2/21)
Total cholesterol (mg/dL)	≤ 200	19% (11/59)	21% (7/33)	19% (4/21)
Triglycerides (mg/dL)	≤ 200	15% (9/59)	6% (2/33)	29% (6/21)
hsCRP (mg/L)	≤ 2	51% (29/57)	61% (20/33)	57% (12/21)
Glucose (mg/dL)	≤ 100	20% (12/59)	18% (6/33)	52% (11/21)
Creatinine (mL/min)	≥ 88	45% (26/58)	41% (14/34)	43% (9/21)
SBP (mmHg)	≤ 130	28% (17/61)	32% (11/34)	45% (10/22)
DBP (mmHg)	≤ 80	13% (8/61)	9% (3/34)	14% (3/22)

BMI: body mass index; ALT: alanine aminotransferase; AST: aspartate aminotransferase; LDL: low-density lipoprotein cholesterol; HDL: high-density lipoprotein cholesterol; hsCRP: high-sensitivity C-reactive protein; SBP: systolic blood pressure; DBP: diastolic blood pressure

## References

[1] O’Bryan RM, Luce JK, Talley RW, Gottlieb JA, Baker LH, Phase II evaluation of adriamycin in human neoplasia. Cancer 1973;32:1–8. 4716773 10.1002/1097-0142(197307)32:1<1::aid-cncr2820320101>3.0.co;2-x

[2] Benjamin RS, Wiernik PH, Bachur NR. Adriamycin chemotherapy--efficacy, safety, and pharmacologic basis of an intermittent single high-dosage schedule. Cancer 1974;33:19–27. 4810094 10.1002/1097-0142(197401)33:1<19::aid-cncr2820330107>3.0.co;2-m

[3] Von Hoff DD, Rozencweig M, Layard M, Slavik M, Muggia FM. Daunomycin-induced cardiotoxicity in children and adults: a review of 110 cases. Am J Med 1977;62:200–8. 835599 10.1016/0002-9343(77)90315-1

[4] Zalupski M, Metch B, Balcerzak S, Fletcher WS, Chapman R, Phase III comparison of doxorubicin and dacarbazine given by bolus versus infusion in patients with soft-tissue sarcomas: a Southwest Oncology Group study. J Natl Cancer Inst 1991;83:926–32. 2067035 10.1093/jnci/83.13.926

[5] Oeffinger KC, Mertens AC, Sklar CA, Kawashima T, Hudson MM, Chronic health conditions in adult survivors of childhood cancer. N Engl J Med 2006;355:1572–82. 17035650 10.1056/NEJMsa060185

[6] Armstrong GT, Liu Q, Yasui Y, Neglia JP, Leisenring W, Late mortality among 5-year survivors of childhood cancer: a summary from the Childhood Cancer Survivor Study. J Clin Oncol 2009;27:2328–38. 19332714 10.1200/JCO.2008.21.1425 PMC2677921

[7] Armenian SH, Armstrong GT, Aune G, Chow EJ, Ehrhardt MJ, Cardiovascular disease in survivors of childhood cancer: insights into epidemiology, pathophysiology, and prevention. J Clin Oncol 2018;36:2135–44. 29874141 10.1200/JCO.2017.76.3920 PMC6804893

[8] Gibson TM, Mostoufi-Moab S, Stratton KL, Leisenring WM, Barnea D, Temporal patterns in the risk of chronic health conditions in survivors of childhood cancer diagnosed 1970–99: a report from the Childhood Cancer Survivor Study cohort. Lancet Oncol 2018;19:1590–601. 30416076 10.1016/S1470-2045(18)30537-0 PMC6309183

[9] Bagnasco F, Caruso S, Andreano A, Valsecchi MG, Jankovic M, Late mortality and causes of death among 5-year survivors of childhood cancer diagnosed in the period 1960–1999 and registered in the Italian Off-Therapy Registry. Eur J Cancer 2019;110:86–97. 30772657 10.1016/j.ejca.2018.12.021

[10] Chatterjee K, Zhang J, Honbo N, Karliner JS. Doxorubicin cardiomyopathy. Cardiology 2010;115:155–62. 20016174 10.1159/000265166 PMC2848530

[11] Rosenberg SA, De_Vita VT, Lawrence TS. DeVita, Hellman, and Rosenberg’s Cancer: Principles & Practice of Oncology. 11th ed. Lippincott, Williams & Wilkins; 2015.

[12] Chow EJ, Chen Y, Hudson MM, Feijen EAM, Kremer LC, Prediction of ischemic heart disease and stroke in survivors of childhood cancer. J Clin Oncol 2018;36:44–52. 29095680 10.1200/JCO.2017.74.8673 PMC5756324

[13] Armstrong GT, Oeffinger KC, Chen Y, Kawashima T, Yasui Y, Modifiable risk factors and major cardiac events among adult survivors of childhood cancer. J Clin Oncol 2013;31:3673–80. 24002505 10.1200/JCO.2013.49.3205 PMC3804290

[14] Ridker PM, Everett BM, Thuren T, MacFadyen JG, Chang WH, Antiinflammatory therapy with canakinumab for atherosclerotic disease. N Engl J Med 2017;377:1119–31. 28845751 10.1056/NEJMoa1707914

[15] Chen Y, Chow EJ, Oeffinger KC, Border WL, Leisenring WM, Traditional cardiovascular risk factors and individual prediction of cardiovascular events in childhood cancer survivors. J Natl Cancer Inst 2020;112:256–65. 31161223 10.1093/jnci/djz108 PMC7073918

[16] Blaes AH, Shenoy C. Is it time to include cancer in cardiovascular risk prediction tools? Lancet 2019;394:986–8. 31443925 10.1016/S0140-6736(19)31886-0

[17] Ridker PM, MacFadyen JG, Everett BM, Libby P, Thuren T, Relationship of C-reactive protein reduction to cardiovascular event reduction following treatment with canakinumab: a secondary analysis from the CANTOS randomised controlled trial. Lancet 2018;391:319–28. 29146124 10.1016/S0140-6736(17)32814-3

[18] Ridker PM, MacFadyen JG, Thuren T, Everett BM, Libby P, Effect of interleukin-1beta inhibition with canakinumab on incident lung cancer in patients with atherosclerosis: exploratory results from a randomised, double-blind, placebo-controlled trial. Lancet 2017;390:1833–42. 28855077 10.1016/S0140-6736(17)32247-X

[19] Arnett DK, Blumenthal RS, Albert MA, Buroker AB, Goldberger ZD, 2019 ACC/AHA Guideline on the Primary Prevention of Cardiovascular Disease: Executive Summary: A Report of the American College of Cardiology/American Heart Association Task Force on Clinical Practice Guidelines. J Am Coll Cardiol 2019;74:1376–414. 30894319 10.1016/j.jacc.2019.03.009 PMC8344373

[20] Grundy SM, Stone NJ, Bailey AL, Beam C, Birtcher KK, 2018 AHA/ACC/AACVPR/AAPA/ABC/ACPM/ADA/AGS/APhA/ASPC/NLA/PCNA Guideline on the Management of Blood Cholesterol: A Report of the American College of Cardiology/American Heart Association Task Force on Clinical Practice Guidelines. Circulation 2019;139:e1082–143. 30586774 10.1161/CIR.0000000000000625 PMC7403606

[21] Ganz PA, Goodwin PJ. Breast cancer survivorship: where are we today? In: Ganz PA, editor. Improving Outcomes for Breast Cancer Survivors: Perspectives on Research Challenges and Opportunities. Cham: Springer International Publishing; 2015. pp. 1–8.

[22] Jacobs LA, Shulman LN. Follow-up care of cancer survivors: challenges and solutions. Lancet Oncol 2017;18:e19–29. 28049574 10.1016/S1470-2045(16)30386-2

[23] Rokitka DA, Curtin C, Heffler JE, Zevon MA, Attwood K, Patterns of loss to follow-up care among childhood cancer survivors. J Adolesc Young Adult Oncol 2017;6:67–73. 27529650 10.1089/jayao.2016.0023 PMC5824664

[24] Suh E, Daugherty CK, Wroblewski K, Lee H, Kigin ML, General internists’ preferences and knowledge about the care of adult survivors of childhood cancer: a cross-sectional survey. An Intern Med 2014;160:11–7. 10.7326/M13-1941 PMC4337806 24573662

[25] Nathan PC, Daugherty CK, Wroblewski KE, Kigin ML, Stewart TV, Family physician preferences and knowledge gaps regarding the care of adolescent and young adult survivors of childhood cancer. J cancer Surviv 2013;7:275–82. 23471729 10.1007/s11764-013-0271-0

[26] Bobowski NP, Baker LH. The university of michigan sarcoma survivorship clinic: preventing, diagnosing, and treating chronic illness for improved survival and long-term health. J Adolesc Young Adult Oncol 2016;5:211–4. 27116634 10.1089/jayao.2016.0004 PMC5076479

[27] D’Agostino RB, Vasan RS, Pencina MJ, Wolf PA, Cobain M, General cardiovascular risk profile for use in primary care: the framingham heart study. Circulation 2008;117:743–53. 18212285 10.1161/CIRCULATIONAHA.107.699579

[28] Ridker PM, Paynter NP, Rifai N, Gaziano JM, Cook NR. C-reactive protein and parental history improve global cardiovascular risk prediction: the Reynolds Risk Score for men. Circulation 2008;118:2243–51. 18997194 10.1161/CIRCULATIONAHA.108.814251 PMC2752381

[29] Orimoloye OA, Mirbolouk M, Uddin SMI, Dardari ZA, Miedema MD, Association between self-rated health, coronary artery calcium scores, and atherosclerotic cardiovascular disease risk: the multi-ethnic study of atherosclerosis (MESA). JAMA Netw Open 2019;2:e188023. 30768193 10.1001/jamanetworkopen.2018.8023 PMC6484585

[30] Harris PA, Taylor R, Thielke R, Payne J, Gonzalez N, Research electronic data capture (REDCap)--a metadata-driven methodology and workflow process for providing translational research informatics support. J Biomed Inform 2009;42:377–81. 18929686 10.1016/j.jbi.2008.08.010 PMC2700030

[31] Armenian SH, Xu L, Ky B, Sun C, Farol LT, Cardiovascular disease among survivors of adult-onset cancer: a community-based retrospective cohort study. J Clin Oncol 2016;34:1122–30. 26834065 10.1200/JCO.2015.64.0409 PMC7357493

[32] Bhagat A, Kleinerman ES. The role of the innate immune system in doxorubicin-induced cardiotoxicity. Am Assoc Immnol 2019;202:187.16.

